# Supplementary material for: Functional reallocation of sensory processing resources caused by long-term neural adaptation to altered optics
Source: eLife. 2021 Feb 22;10:e58734. doi: 10.7554/eLife.58734 (PMC7963487; doi:10.7554/eLife.58734)
Supplement: Transparent reporting form [file elife-58734-transrepform.pdf]

eLIFE
1st Floor
24 Hills Road
Cambridge CB2 1JP, UK
P 01223 855340
W elifesciences.org
T @elife

# *eLife's* transparent reporting form

We encourage authors to provide detailed information *within their submission* to facilitate the interpretation and replication of experiments. Authors can upload supporting documentation to indicate the use of appropriate reporting guidelines for health-related research (see EQUATOR Network), life science research (see the BioSharing Information Resource), or the ARRIVE guidelines for reporting work involving animal research. Where applicable, authors should refer to any relevant reporting standards documents in this form.

If you have any questions, please consult our Journal Policies and/or contact us: editorial@elifesciences.org.

**Sample-size estimation**

- You should state whether an appropriate sample size was computed when the study was being designed
- You should state the statistical method of sample size computation and any required assumptions
- If no explicit power analysis was used, you should describe how you decided what sample (replicate) size (number) to use

Please outline where this information can be found within the submission (e.g., sections or figure legends), or explain why this information doesn't apply to your submission:

Sample size is indicated several times within the manuscript (Results Section, Methods–participants), and in all Figures showing results. Sample size was determined based on previously published studies that used adaptive optics to study visual perception (e.g., Artal et al., 2004; Sabesan & Yoon 2009; 2010; Zheleznyak et al., 2016). A-priori power analysis was not possible as there was no reasonable way to estimate the effect size.

**Replicates**

- You should report how often each experiment was performed
- You should include a definition of biological versus technical replication
- The data obtained should be provided and sufficient information should be provided to indicate the number of independent biological and/or technical replicates
- If you encountered any outliers, you should describe how these were handled
- Criteria for exclusion/inclusion of data should be clearly stated
- High-throughput sequence data should be uploaded before submission, with a private link for reviewers provided (these are available from both GEO and ArrayExpress)

Please outline where this information can be found within the submission (e.g., sections or figure legends), or explain why this information doesn't apply to your submission:

eLife Sciences Publications, Ltd is a limited liability non-profit non-stock corporation incorporated in the State of Delaware, USA, with company number 5030732, and is registered in the UK with company number FC030576 and branch number BR015634 at the address 1st Floor, 24 Hills Road, Cambridge CB2 1JP | August 2014

All information regarding the amount of data collected is detailed in the Material and Methods for each experiment. No data was excluded. As indicated in the manuscript (e.g., p.8), the results of the visual acuity (VA) experiment replicated previous findings (Sabesan & Yoon, 2009). Multiple threshold estimates were collected for each observer, with low variability observed within a given observer. For the qCSF experiment, we collected multiple qCSF runs for each observer to reliably estimate contrast sensitivity across a wide spatial frequency (SF) range. The reliability and efficiency of the qCSF method has been well characterized (e.g., Lesmes et al., 2010; Hou et al., 2010; 2016). Moreover, as mentioned (p.12), the results of the equivalent noise paradigm experiment replicated the main findings of the qCSF experiment (i.e., impaired contrast sensitivity at high SFs and better sensitivity at low SFs), despite differences in stimulus and experimental design. Finally, we also indicate that our results replicate previous studies (e.g., Sabesan et al., 2007; Sabesan et al., 2016; Sabesan & Yoon, 2009; Sabesan, Zheleznyak & Yoon, 2012; Zheleznyak et al., 2016) showing that adaptive optics correction can effectively maintain aberration-free optical quality during visual testing (p.4, 20).

**Statistical reporting**

- Statistical analysis methods should be described and justified
- Raw data should be presented in figures whenever informative to do so (typically when N per group is less than 10)
- For each experiment, you should identify the statistical tests used, exact values of N, definitions of center, methods of multiple test correction, and dispersion and precision measures (e.g., mean, median, SD, SEM, confidence intervals; and, for the major substantive results, a measure of effect size (e.g., Pearson's r, Cohen's d)
- Report exact p-values wherever possible alongside the summary statistics and 95% confidence intervals. These should be reported for all key questions and not only when the p-value is less than 0.05.

Please outline where this information can be found within the submission (e.g., sections or figure legends), or explain why this information doesn't apply to your submission:

Individual estimates are shown for each experiment (e.g., Fig.2,4,5,7,8). N is indicated several times within the manuscript (e.g., Results, Methods Section – Participants), and in each Figure showing results. Averaged estimates are indicated along with a dispersion measure (SD, SEM or 95%-CI) in the Results section and in the Figures. Statistical tests are specified within the Results and Methods sections, and a measure of effect size is provided for each test (Cohen's d, partial eta-squared, Pearson's r, rank biserial correlation). The exact p-value is always reported, except for values less than 0.001. In the qCSF experiment, comparisons at each of the 12 spatial frequency levels were corrected for multiple comparisons using Bonferroni correction, as indicated in the Figures and Methods. Assumptions of normality and of equality of variance were tested using the Shapiro-Wilk test and the Levene's test, respectively, and appropriate statistical testing was used if needed as indicated in the Methods section (e.g., p.20).

(For large datasets, or papers with a very large number of statistical tests, you may upload a single table file with tests, Ns, etc., with reference to sections in the manuscript.)

**Group allocation**

eLife Sciences Publications, Ltd is a limited liability non-profit non-stock corporation incorporated in the State of Delaware, USA, with company number 5030732, and is registered in the UK with company number FC030576 and branch number BR015634 at the address 1st Floor, 24 Hills Road, Cambridge CB2 1JP | August 2014

- Indicate how samples were allocated into experimental groups (in the case of clinical studies, please specify allocation to treatment method); if randomization was used, please also state if restricted randomization was applied
- Indicate if masking was used during group allocation, data collection and/or data analysis

Please outline where this information can be found within the submission (e.g., sections or figure legends), or explain why this information doesn't apply to your submission:

All observers were screened by one of our ophthalmologists to ensure they could participate in our study (see Methods – Participants, p.19). Patients diagnosed with keratoconus were allocated to the keratoconus group, and observers with typical 'healthy' optics were allocated to the typical-control group. No masking was used.

**Additional data files ("source data")**

- We encourage you to upload relevant additional data files, such as numerical data that are represented as a graph in a figure, or as a summary table
- Where provided, these should be in the most useful format, and they can be uploaded as "Source data" files linked to a main figure or table
- Include model definition files including the full list of parameters used
- Include code used for data analysis (e.g., R, MatLab)
- Avoid stating that data files are "available upon request"

Please indicate the figures or tables for which source data files have been provided:

All data will be made available on OSF and indicated in the manuscript before publication of the final version.
